# Unveiling the Effects of Hydrolysis‐Derived DMAI/DMAPbI*_x_* Intermediate Compound on the Performance of CsPbI_3_ Solar Cells

**DOI:** 10.1002/advs.201902868

**Published:** 2020-03-14

**Authors:** Hui Bian, Haoran Wang, Zhizai Li, Faguang Zhou, Youkui Xu, Hong Zhang, Qian Wang, Liming Ding, Shengzhong (Frank) Liu, Zhiwen Jin

**Affiliations:** ^1^ Key Laboratory of Applied Surface and Colloid Chemistry Shaanxi Key Laboratory for Advanced Energy Devices Shaanxi Engineering Lab for Advanced Energy Technology School of Materials Science & Engineering Ministry of Education Shaanxi Normal University Xi'an 710119 P. R. China; ^2^ School of Physical Science and Technology & Key Laboratory for Magnetism and Magnetic Materials of MoE & Key Laboratory of Special Function Materials and Structure Design MoE & National & Local Joint Engineering Laboratory for Optical Conversion Materials and Technology Lanzhou University Lanzhou 730000 P. R. China; ^3^ Electron Microscopy Centre School of Physical Science and Technology Lanzhou University Lanzhou 730000 P. R. China; ^4^ Center for Excellence in Nanoscience (CAS) Key Laboratory of Nanosystem and Hierarchical Fabrication (CAS) National Center for Nanoscience and Technology Beijing 100190 P. R. China

**Keywords:** CsPbI_3_, DMAI, DMAPbI*_x_*, intermediate compounds, solar cells

## Abstract

Introducing hydroiodic acid (HI) as a hydrolysis‐derived precursor of the intermediate compounds has become an increasingly important issue for fabricating high quality and stable CsPbI_3_ perovskite solar cells (PSCs). However, the materials composition of the intermediate compounds and their effects on the device performance remain unclear. Here, a series of high‐quality intermediate compounds are prepared and it is shown that they consist of DMAI/DMAPbI*_x_*. Further characterization of the products show that the main component of this system is still CsPbI_3_. Most of the dimethylammonium (DMA^+^) organic component is lost during annealing. Only an ultrasmall amount of DMA^+^ is doped into the CsPbI_3_ and its structure is stabilized. Meanwhile, excessive DMA^+^ forms Lewis acid–base adducts and interactions with Pb^2+^ on the CsPbI_3_ surface. This process passivates the CsPbI_3_ film and decreases the recombination rate. Finally, CsPbI_3_ film is fabricated with high crystalline, uniform morphology, and excellent stability. Its corresponding PSC exhibits stable property and improved power conversion efficiency (PCE) up to 17.3%.

## Graphical abstract

This work mainly focuses on materials composition and working mechanism of the hydroiodic acid (HI) hydrolysis‐derived intermediate compound DMAI/DMAPbI*_x_*. Importantly, the main component of the CsPbI_3_ film prepared by such precursor is proved to be still inorganic. Finally, the optimized CsPbI_3_ film–based device shows significantly enhanced stability in ambient environment with a high power conversion efficiency of 17.32%.

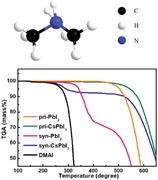

Compared with the emerging photovoltaic materials, halide perovskites with tunable bandgaps, long carrier diffusion, high defect tolerance, superior optical absorption coefficients, and low exciton binding energies are becoming promising materials for solar cells.^1^, ^2^, ^3^, ^4^, ^5^, ^6^ Due to the intrinsic thermal and light instabilities of cation A in hybrid organic‐inorganic halide perovskite materials, Cs^+^ is the most widely used inorganic cation in the perovskite structure (ABX_3_) for making up those shortcoming.^7^, ^8^, ^9^, ^10^, ^11^, ^12^ Among numerous all‐inorganic perovskites, CsPbI_3_ has highly efficient optical, electrical properties and appropriate bandgap (*E*_g_ = 1.7 eV), which is much ideal for photovoltaic applications.^13^, ^14^, ^15^, ^16^, ^17^, ^18^

However, black phase‐based CsPbI_3_ is not stable and degrades rapidly to an undesired yellow phase δ‐CsPbI_3_ in air (*E*_g_ = 2.82 eV).^19^, ^20^, ^21^, ^22^, ^23^ Many efforts have been implemented on CsPbI_3_ to improve its structure stability via solution‐processed methods especially used a facile additive engineering.^24^, ^25^, ^26^, ^27^ Though hydroiodic acid (HI) additive can improve the stability and efficiency of inorganic perovskite solar cells (PSCs), the complete mechanism is unclear.^28^, ^29^

Researchers initially used HI as a reliable nonstoichiometric acid–base reaction route to modulate the crystal structure and optoelectronic properties of PSCs.^30^ Snaith and coworkers first stabilized the CsPbI_3_ films in the black phase at room temperature via a HI additive. This made grain refinement and obtained the highest stabilized power conversion efficiency (PCE) of 1.7%.^31^ They believed the greater phase stability was caused by strain in the crystals which induced by the HI additive. Later, Zhao et al. stated that the polar solvent (actually the water in the HI) affects ionization in the precursor solution leading to a smaller crystal size and stabilized γ‐phase.^32^, ^33^

In 2015, researchers reported a new precursor compound hydrogen lead iodide (HPbI_3_), fabricated by adding HI into PbI_2_ and dimethyl formamide (DMF) precursor solution, as intermediate compound for hybrid perovskite solar cells.^34^, ^35^, ^36^ Zhao and coworkers first used HPbI_3_ to fabricate CsPbI_3_ film with much enhanced PCE and stability.^37^, ^38^ Chen and coworkers discovered highly stable α‐CsPbI_3_ with a reduced bandgap (1.68 eV) can be obtained in dry air by replacing PbI_2_ with HPbI_3_.^39^ In our early work, we also believed that the HPbI_3_ replaces PbI_2_ leading to the formation of a stabilized distorted black phase.^40^ Most recently, Xi et al. increased HI amount to fabricate another new compound H_2_PbI_4_ to fabricate stabilized CsPbI_3_ PSCs.^41^

In 2018, G. Kanatzidis and coworkers realized that HPbI_3_ is actually the hybrid perovskite cesium dimethylammonium lead iodide (Cs_1−_ *_X_*DMA*_X_*PbI_3_). In fact, inorganic perovskites are still hybrid organic‐inorganic perovskites.^42^ Here, HI acts with PbI_2_ containing DMF to remove H_2_O, PbO, and PbO_2_ in PbI_2_ and also eventually form dimethylammonium (DMA^+^) group.^43^ Similarly, Liu and coworkers reported hydrolysis‐derived materials (i.e., DMAPbI_3_) and analyzed its role in producing high‐quality PSCs in detail by changing the ratio of CsI/DMAPbI_3_ in the precursor.^44^ However, Zhao and coworkers established that an optimized annealing process could remove all organic species. This ruled out the incorporation of the organic DMA^+^ A‐site cations.^45^

Thus, the materials composition of the intermediate compound is still unclear. The corresponding CsPbI_3_ films likely contain an organic group. The effect and the mechanism of DMA^+^‐enhanced and stabilized device performance are still a mystery. Hence, we mainly focus on the materials composition of HI hydrolysis‐derived intermediate compound (DMAI/DMAPbI*_x_*) by adjusting the volumes of HI. Next, we analyzed whether the synthetic CsPbI_3_ film (fabricated by DMAI/DMAPbI*_x_* precursor) contains organic groups and proved the major component is still inorganic in this reaction route. Finally, we systematically evaluated the effect of DMAI/DMAPbI*_x_* on CsPbI_3_ PSCs performance.

Here, the hydrolysis‐derived intermediate compound (marked as syn‐PbI_2_) was synthesized by adding the HI to PbI_2_ and DMF mixed solution followed by thermal stirring at 80 °C for 120 min (**Figure** **1**a). The DMAI powders were similarly synthesized by adding the HI to pure DMF solution (Figure 1b). The color changes from dark yellow (pristine PbI_2_, marked as pri‐PbI_2_) to light yellow as the reaction proceeds. Here, the volume ratio of DMF:HI is precisely changed (DMF:HI = 1:1, 1:2, 1:3, and 1:4 v/v); all samples had the same purification technique. The details are described in the Experimental Section.

**Figure 1:**
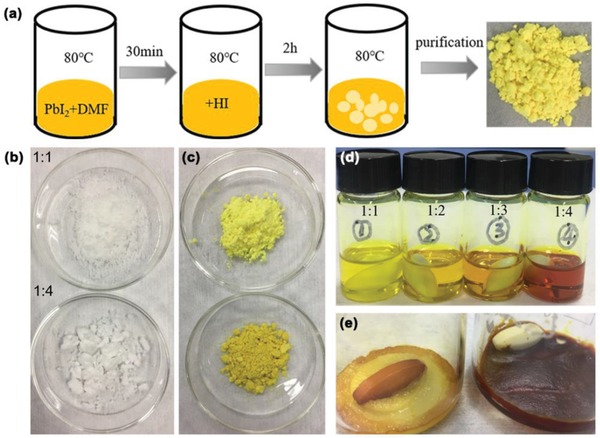
Synthesis and characterization of the Syn‐PbI_2_ and DMAI Powder. a) Synthesis. A series of photographs of b) DMAI powder and c) syn‐PbI_2_ powder. d) Precursor solution prepared using different HI ratios and stored for 1 month. e) Unpurified crystalline solid of DMAI.

The HI content may directly affect the quality of syn‐PbI_2_ powder: The color of syn‐PbI_2_ powder prepared with a larger HI ratio is slightly darker than those made at lower ratios (Figure 1c). The large differences in the color and viscosity for the solution and products are likely due to different volume radio of DMF:HI. Figure 1d shows the precursor solution prepared with different syn‐PbI_2_ powders after one month of storage. The color of the solution begins to deepen with increasing HI dosage due to its larger iodine content. Figure 1e shows the unpurified crystal solid of DMAI. It shows more HI addition causing the reaction solution to be darker, resulting in the final solid darker. However, after being purified by diethyl ether, the pure DMAI was obtained.

In order to deeply comprehend the properties of syn‐PbI_2_ powder and possible influence on the corresponding CsPbI_3_ films (marked as syn‐CsPbI_3_), next, we used scanning electron microscope (SEM) images and energy‐dispersive spectrometric (EDS) mapping to further study the materials. **Figure** **2**a shows the molecular structure of DMA^+^ (2.72 Å) cations. These belong to organic groups and have a very large radius versus Cs^+^ (1.88 Å).^46^ Figure 2b shows SEM image of syn‐PbI_2_. This has a deformed hexagonal rod‐like shape. Figure 2c–g reveals that N, Pb, and I are uniformly overlaid, and corresponding EDS mapping shows Pb, I, C, and N. The syn‐PbI_2_ powder is probably DMAI/DMAPbI*_x_* powder.

**Figure 2:**
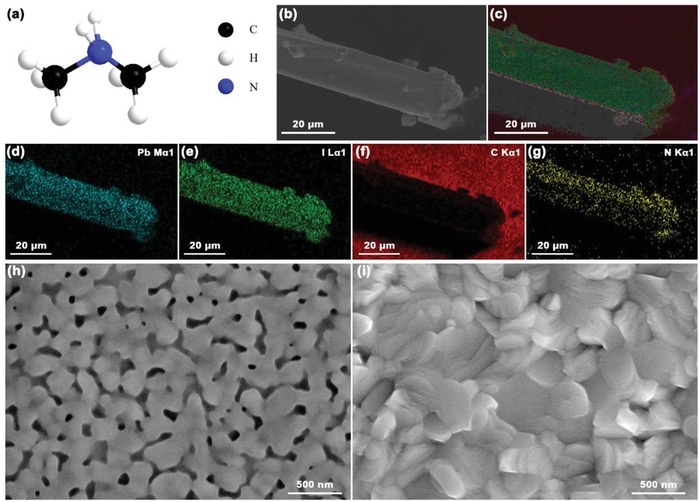
Characterization of the Syn‐PbI_2_ Powder and the Corresponding Syn‐CsPbI_3_ film. a) Molecular structures of DMA^+^; b) SEM image; c) EDS mapping of N, Pb, and I; d–g) EDS mapping of d) Pb element, e) I element, f) C element, and g) N element distribution; and SEM image of h) unannealed and i) annealed syn‐CsPbI_3_ film.

We also collected images of the unannealed and annealed syn‐CsPbI_3_ perovskite film (Figure 2h,i, respectively). The syn‐CsPbI_3_ film without annealing obviously has many pores and cracks that are relatively uniformly distributed. After annealing, the film surface is fully covered with rough morphology. We also provided the SEM image (Figure S1a,d, Supporting Information) and the corresponding EDX spectra of point A, B, C, and D of unannealed and annealed syn‐CsPbI_3_ film in Figure S1 in the Supporting Information. Figure S1b,c in the Supporting Information shows that the N content of the unannealed film is necessarily present, although there is a certain measurement error. After annealing for a certain time, there is a very small amount of N element, or even almost no, respectively in Figure S1e,f in the Supporting Information. In order to reduce the error, we performed a surface scan of unannealed and annealed syn‐CsPbI_3_ film as shown in Figure S2 in the Supporting Information. The EDS shows that N content decreases from 5.6% before annealing to 2.2% after annealing. This suggests that PbI_2_ forms Lewis adducts with DMA^+^ that compete with the inorganic cations (Cs in this report). However, the Cs^+^ combined with the Pb^2+^ more tightly than DMA^+^, and many organic components escape during the annealing process.

We further determine the role of HI ratio on the preparation of syn‐PbI_2_ powder and the nature of the syn‐CsPbI_3_ films. **Figure** **3**a shows X‐ray diffraction (XRD) patterns and corresponding magnifications of a portion of the XRD patterns for syn‐PbI_2_ powders with varying amounts of HI. The peak of PbI_2_ at 12.8° corresponds to a layered structure with an interlayer spacing of 6.98 Å along the z‐axis.^34^ The syn‐PbI_2_ adopts a similar XRD pattern with a low angle peak shifted to 11.6°, indicating an expansion of the interlayer spacing originating from the intercalation of DMA^+^ ions. The shift of peak position toward a small angle direction along with the HI amount may be associated with the expanded lattice volume, which caused by excessing iodide and excessive DMA^+^ ion. With HI added, the DMA^+^ ions are doped into the lattice during the iodide rich environment. Meanwhile, the peak intensities of the XRD are significantly improved as the HI content increases indicating that the crystallinity is greatly enhanced by excessive DMA^+^ ion. The XRD pattern for DMAI powder show peaks at 17.16, 19.68, 23.49, 24.79, 26.62, 28.28, and 34.7, which can be well ascribed to the purchased and reported DMAI (Figure S3a, Supporting Information).^42^ In addition, in order to compare the effect of different ratios of HI on the preparation of DMAI (guaranteeing the same amount of solvent used for purification). The XRD pattern of the 1:4 syn‐DMAI exhibits a lattice expansion, with a 0.2° shift to lower angle compared to 1:1 syn‐DMAI (Figure S3b,c, Supporting Information), which is caused by the existing of negative iodine system and the filling of larger I^−^ ion.

**Figure 3:**
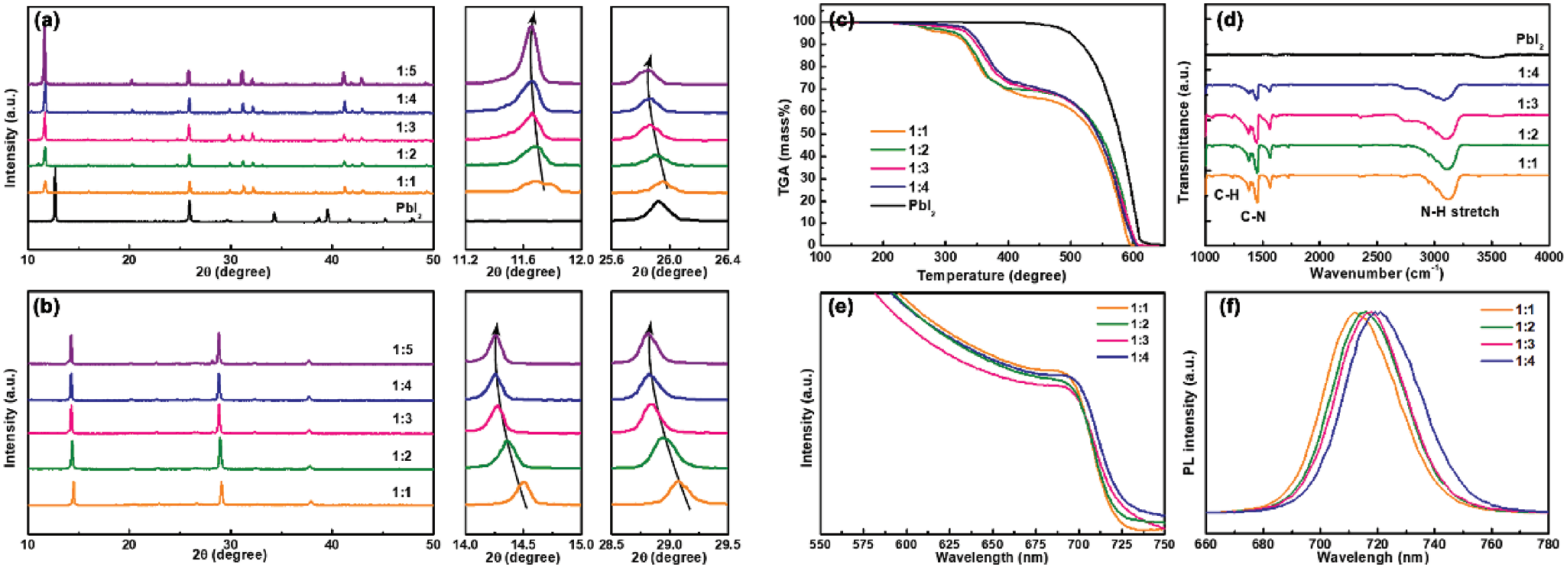
Characterization of Syn‐PbI_2_ powder and Syn‐CsPbI_3_ films prepared using different HI ratios. XRD patterns and the corresponding magnification of a portion of the XRD patterns of a) syn‐PbI_2_ powder and b) syn‐CsPbI_3_ films. c) TGA spectra of syn‐PbI_2_ powder. d) FTIR spectra of syn‐PbI_2_ powder. e) Optical absorption spectra of syn‐CsPbI_3_ films and f) PL spectra of syn‐CsPbI_3_ films.

For the syn‐PbI_2_, the dominant diffraction peaks of standard XRD pattern of DMAPbI_3_ were observed (Figure S4a, Supporting Information), which corresponds to the peaks of 11.6°, 20.2°, 25.8°, 29.8°, 31.2°, 32.2°, 41.1°, and 42.9°, respectively. In addition to DMAPbI_3_ peaks, other main peaks at 24.6°, 33.1°, and 35.0° are matching well with corresponding planes of DMAI. Importantly, no others impurities peaks remained except DMAPbI_3_ and DMAI for syn‐PbI_2_, implying the highly purity for the DMAI/DMAPbI*_x_* composites. Besides, Figure S4b in the Supporting Information shows the syn‐PbI_2_ exhibits a shift to low angle compared to the corresponding magnification of a portion of XRD pattern for DMAPbI*_x_*, indicating an expanded lattice volume originating from adding excessing iodide. Therefore, we believe that adding excessing iodide will promote the formation of negative iodine system in DMAPbI*_x_*. As aforementioned discussion, we proved the syn‐PbI_2_ powder is DMAI/DMAPbI*_x_* powder.

The fabricated syn‐CsPbI_3_ films exhibit Bragg peaks at 14.33° and 28.89° that can be assigned to the (110) and (220) planes of the CsPbI_3_ crystallites (Figure 3b).^47^ We calculated the full width at half maximum (FWHM) of the main peaks as shown in Figure S5 in the Supporting Information. There is a greater effect on the crystallinity of syn‐PbI_2_ powders as the increase in HI content. However, the effect on the crystallinity of the syn‐CsPbI_3_ film is small, indicating that the change in HI content has little effect on the particle size of the crystallization process. Not surprisingly, the peak shift of syn‐CsPbI_3_ film has a similar trend with the syn‐PbI_2_ powders. This is likely due to the DMA^+^ ion doped into the CsPbI_3_ to partly replace the Cs^+^ and expand the lattice.^48^, ^49^ Furthermore, DMAPbI*_x_* prepared by excessing iodide also causes the lattice volume more larger, and during annealing, I^−^ ion is not easily decomposed and remains in the crystal lattice. Above all, although the composition for syn‐CsPbI_3_ films and syn‐PbI_2_ powders with different DMF:HI ratios is basically similar, its show a noticeable effect on the phase transitions and optoelectronic because of soft mechanical nature of the hybrid perovskites.^50^ Further increases HI amount (1:5) do not change the XRD patterns of the syn‐CsPbI_3_ film. This probably reason is that the syn‐PbI_2_ powder prepared by superfluous HI has a stable component (DMAPbI*_x_*). These data indicate that the optimal DMF/HI volume ratio is 1:4. Therefore, the studies below use 1:1, 1:2, 1:3, and 1:4 syn‐PbI_2_ powder for the corresponding syn‐CsPbI_3_ films.

To examine the thermal stability of the syn‐PbI_2_ powder, thermogravimetric analysis (TGA) and differential scanning calorimetry (DSC) is performed in nitrogen as shown in Figure 3c and Figure S6 in the Supporting Information, respectively. Confusing thermal behavior is seen in syn‐PbI_2_ TGA curves. Three thermal events occur in all samples with 100% weight loss. The DMA group has stronger bonding with Pb atoms, and DMAPbI*_x_* shows enhanced thermal stability versus DMAI. The material with a DMF/HI volume ratio of 1:1 first undergoes a ≈4% mass loss of DMAI at 277 °C followed by a ≈26% loss of the organic ligands (DMAI) and ≈70% loss of PbI*_x_* at 315 and 605 °C, respectively. Correspondingly, the exothermic peaks are observed in the DSC curves when the DMAI lost. Similar component decomposition is seen for 1:2, 1:3, and 1:4 samples except that their thermal behaviors show a slightly gradual change. The calculations (**Table** **1**) show that the molar ratio of DMAPbI*_x_* in syn‐PbI_2_ powder changed from 96% to 99.3%, leading to an iodine‐rich system (*x* changed from 3.04 to 3.86 in DMAPbI*_x_*) by adding excess HI. These results suggest that the transformation process can be written as

(1) $${\mathrm{PbI}}_{2}\;+\;\mathrm{DMF}\;+\;\mathrm{HI}\;\to \;{\mathrm{syn-PbI}}_{2}\left(\mathrm{DMAI}\;+\;{\mathrm{DMAPbI}}_{x}\right)$$

(2) $${\mathrm{syn-PbI}}_{2}\left(\mathrm{DMAI}\;+\;{\mathrm{DMAPbI}}_{x}\right)\;\to \;{\mathrm{DMAPbI}}_{x}\;+\;\mathrm{DMAI}↑\left(g\right)$$

(3) $${\mathrm{DMAPbI}}_{x}\;\to \;{\mathrm{PbI}}_{x-1}\;+\;\mathrm{DMAI}↑\left(g\right)$$

**Table 1:** TGA parameters of different syn‐PbI_2_ powders (extracted from Figure 3c)

Sample	DMAI			DMAPbI*_x_*			
	Temperature [°C]	*Δ* mass [%]	Ratio [%]	Temperature [°C]	*Δ* mass [%]	Ratio [%]	*x*
1:4	246	≈0.7	≈0.7	402	≈23.1	≈99.3	≈3.86
1:3	265	≈1.5	≈1.5	397	≈23.8	≈98.5	≈3.65
1:2	274	≈2.8	≈2.8	387	≈25.7	≈97.2	≈3.16
1:1	277	≈4.0	≈4.0	377	≈26.0	≈96.0	≈3.04

To elucidate the chemical compositions of syn‐PbI_2_ prepared under different conditions, the Fourier transform infrared reflectance (FTIR) spectra are carried out as shown in Figure 3d. The clear signatures of the N—H stretching mode (3250–3480 cm^−1^), C—H bending mode (1230–1260 cm^−1^), and C—N bending mode (1470–1590 cm^−1^) can be ascribed to the presence of the DMA^+^ group.^51^ In addition, Figure S7 in the Supporting Information shows the X‐ray photoelectron spectroscopy (XPS) spectra of the Pb 4f and I 3d5 peaks. The peak positions move to higher binding energy from 1:1 to 1:4 samples.

The XPS data of syn‐CsPbI_3_ films are shown in Figure S8 in the Supporting Information including the Cs 3d5, Pb 4f, Br 3d5, and I 3d5 peaks. The same result on the shift of the peak position is discovered. These results clearly demonstrate that there is strong interaction in syn‐PbI_2_ powder and syn‐CsPbI_3_ film. These data may result from HI of different ratios and are explained by the Pauling electronegativity theory. With the amount of HI increasing, more DMA^+^ atoms are doped, because the pseudo‐alkali metal DMA has a larger electronegativity than Cs; this indicates a negative charge transfer toward DMA^+^ that increases the Pb 4f and I 3d5 core level binding energy.^52^

Next, we determined whether these results are related to the varying content or the purification process. Figure S9 in the Supporting Information shows the FTIR spectrum and TGA of the syn‐PbI_2_ powder washed with either a fixed volume once or three times. As expected, the number of purifications process impacts the content of the final syn‐PbI_2_ powder, but there is no change in chemical composition or properties. Therefore, we quite certain that the reason for the change in the properties of syn‐PbI_2_ powder is the addition of different amounts of HI additive.

Then, we investigated the optoelectronic properties of the syn‐CsPbI_3_ films to gain insight into additive processing and the passivation mechanism. Figure 3e shows the UV–vis spectra. The absorption onset for the syn‐CsPbI_3_ film in the 1:1 sample is at 720 nm corresponding to a bandgap (*E*_g_) of 1.72 eV. However, a similar absorption and slight red shift occur to the 735 nm on side of the absorption band as the HI content increases. The photoluminescence (PL) measurements in Figure 3f also have a red shift from 713 to 722 nm for the 1:1 and 1:4 samples, respectively. The redshifts suggest the formation of narrower bandgaps in both absorption and emission spectra.

In general, *E*_g_ narrowing might be attributed to various reasons, e.g., the grain size effect, phase transition, H‐doping, and lattice strain.^39^ First, the grain size effect may be ruled out because the syn‐films no obvious change in grain size. In addition, as revealed in XRD results, no new CsPbI_3_‐related perovskite phase is detected as the HI content increasing, and therefore, the phase transition is not relevant to the observed *E*_g_ narrowing. It is found that H‐doping would result in metallization of α‐CsPbI_3_ in spite of the H‐doping position, which suggests that H‐doping is not the critical reason in our experiment. Therefore, *E*_g_ narrowing can attribute to tensile lattice strain because of lattice expansion. According to our experimental results, *E*_g_ narrowing can attribute to increased lattice strain because of lattice expansion. Hence, these measurements suggest that increasing HI content may increase the useful organic groups (DMA^+^) doped into the component.^53^

The charge carrier kinetics were further confirmed for the perovskite film on glass substrate via time‐resolved photoluminescence (TRPL, Figure S10 and Table S1, Supporting Information). The average lifetime (τ_ave_) is calculated using the following Equation^54^, ^55^

(4) $$\tau_{\mathrm{ave}}=\frac{\sum A_{i}\tau_{i}^{2}}{\sum A_{i}\tau_{i}}$$

where *A_i_* is the decay amplitude, and *τ_i_* is the decay time. According to the previous studies in perovskites, *τ_1_* corresponds to the fast nonradiative recombination which is induced by defects or impurities introduced during preparation of grains. The slower time constant *τ_2_* represents radiative recombination of the charge carriers.^56^ The τ_ave_ values are 1.56, 4.18, 4.76, and 10.87 ns for the 1:1, 1:2, 1:3, and 1:4 syn‐CsPbI_3_ films, respectively, which confirms that the I‐rich syn‐PbI_2_ fabricated perovskite absorbing layer with lower trap density, better charge separation, and charge extraction.^57^, ^58^

To further confirm the material composition and the interaction of DMAI, optimized syn‐PbI_2_ powder (1:4), pri‐PbI_2_ powder, optimized syn‐CsPbI_3_ (1:4), and pri‐CsPbI_3_ (fabricated by CsI and PbI_2_) films were characterized by XPS (**Figure** **4**a). As expected, the pri‐PbI_2_ powder and pri‐CsPbI_3_ film do not show the N 1s peak. The peak located at 401 eV belongs to N 1s of DMAI, suggesting a different chemical environment versus the syn‐PbI_2_ powder. The XPS spectrum of the syn‐PbI_2_ powder and the syn‐CsPbI_3_ film shows a peak at 402 eV agreeing with N 1s. This indicates the presence of a DMA^+^ group in the syn‐PbI_2_ powder and syn‐CsPbI_3_ film. In addition, the N peak intensity of the syn‐CsPbI_3_ film is significantly lower than the syn‐PbI_2_ powder indicating that the interaction of N is weakened due to the reduced content of the DMAI in the final film after the annealing process (Figure 2).

**Figure 4:**
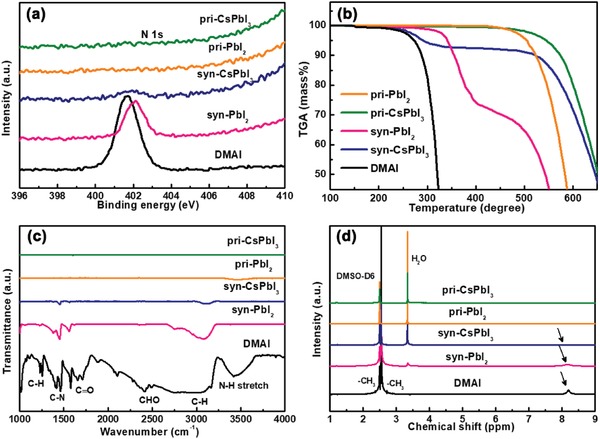
Characteristics of DMAI, Syn‐PbI_2_, Pri‐PbI_2_, Syn‐CsPbI_3_, and Pri‐CsPbI_3_ powders. a) XPS spectra of N 1s; b) TGA curves; c) FTIR spectra; and d) 1H NMR spectra (the powders for NMR measurement were dissolved in DMSO‐d6).

Figure 4b shows the TGA curves of individual components. The thermal stability of DMAI is poor, and thermal decomposition occurs below 200 °C. The Pri‐PbI_2_ and pri‐CsPbI_3_ powder have direct sublimation without complex decomposition at the onset temperatures of 465 °C. The syn‐PbI_2_ film tends to decompose from 250 to 350 °C suggesting that large amounts of organic ligands are prone to rapid decomposition. The decomposition of syn‐CsPbI_3_ has a similar trend as the DMAI powder. The Cs^+^ is likely combined with the Pb more tightly than DMA^+^ during the fabrication process. This indicates that the syn‐CsPbI_3_ film consists of Cs_1−_ *_x_*DMA*_x_*PbI_3_, and the syn‐CsPbI_3_ film can be purified due to decomposition of Cs_1−_ *_x_*DMA*_x_*PbI_3_ to DMAI and CsPbI_3_.

To get more accurate information about material composition, we next examine their functional groups by FTIR (Figure 4c). There is a distinct peak at 3480 cm^−1^ in DMAI, which belongs to the N—H vibration mode. However, the syn‐PbI_2_ powder and syn‐CsPbI_3_ film do not have this peak; rather they exhibit other peaks in the organic groups belonging to the characteristic peaks of N—H, C—N, and C—H. No similar phenomenon is observed in pri‐PbI_2_ powder and pri‐CsPbI_3_ film. The infrared peak strength of DMAI, syn‐PbI_2_ powder, and syn‐CsPbI_3_ film is gradually weakened indicating that most of the organic compounds escape after 180 °C annealing treatment.^45^

Figure 4d shows ^1^H nuclear magnetic resonance (NMR) spectroscopy data: The distinct signals at *d* = 2.55 and 8.15 ppm belong to protons in CH_3_— and —NH_2_—, respectively. The peak intensity of —NH_2_— is very weak and is barely detected in the syn‐CsPbI_3_ film consistent with FTIR test. In addition, the main component of syn‐CsPbI_3_ is still inorganic. Notably, these results demonstrate that there is an enormous difference between pri‐CsPbI_3_ and syn‐CsPbI_3_ perovskite films prepared with different precursor.

SEM (**Figure** **5**a) and atomic force microscopy (AFM, Figure 5b) suggested that syn‐CsPbI_3_ films prepared from different pre‐PbI_2_ precursors are all compact and uniform with no obvious differences in grain size. EDS showed that N, Cs, Pb, I, Br, and C were uniformly distributed on the surface (Figure 5c). These results strongly indicate the existence and distribution of DMA^+^ on the surface of syn‐CsPbI_3_ film. At a depth of 10 nm, the N/Pb atomic ratio drops dramatically from 0.5% to 0%. This shows that the film is fabricated by CsPbI_3_ and the surface is strongly enriched with DMA^+^ (Figure S11 and Table S2, Supporting Information).

**Figure 5:**
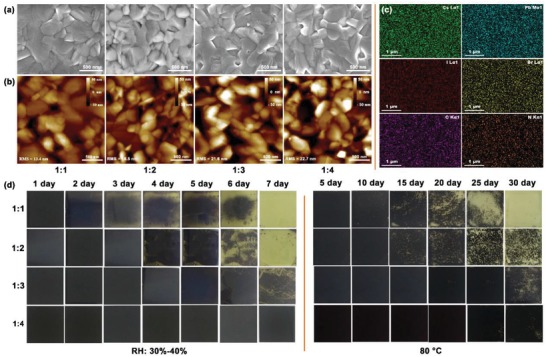
Morphology characterization of Syn‐CsPbI_3_ films prepared using different HI ratios. a) SEM images; b) AFM images; c) EDS mapping for Cs, Pb, I, Br, C, and N elements. d) Images of syn‐CsPbI_3_ films exposed to air at different durations with 30–40% RH or in a glove box at 80 °C.

The phase instability of pure‐CsPbI_3_ restricts the light harvest and charge transport efficiency because of spontaneous degradation under ambient and temperature. Therefore, we traced photographs of syn‐CsPbI_3_ films at different durations of exposure to air with 30–40% RH in N_2_ or in a glove box at 80 °C (Figure 5d). The results show that the film prepared using superabundant HI can maintain its black phase structure for up to 7 days in air with 30–40% RH. In contrast, the syn‐CsPbI_3_ films using precursor preparations with less HI are more susceptible to fading from black to yellow over time; they are almost totally bleached after 7 days. As expected, these syn‐CsPbI_3_ films of different precursors have the same air stability as the sample in the glove box at 80 °C. The 1:4 sample is more stable than the 1:1 sample. It has a delayed degradation reaction, which might be due to the small amount of DMA^+^ doped into the CsPbI_3_ that adjusts its crystal structure.^59^, ^60^

Finally, we evaluated the photovoltaic performance using a typical normal cell structure (glass substrate/fluorine‐doped tin oxide (FTO)/TiO_2_/CsPbI_3_/Poly[bis(4‐phenyl) (2,4,6‐trimethylphenyl)amine] (PTAA)/Au).^40^ The *J*–*V* curve of the devices based on syn‐CsPbI_3_ films is presented in **Figure** **6**a, and specific photovoltaic parameters are shown in **Table** **2**. Obviously, the cell performance is significantly improved as the HI content increases. It has an outstanding PCE of 17.3%. We processed a performance comparison between the 1:1 and 1:4 syn‐CsPbI_3_ PSCs with the DMAI completely removed after longer annealing process (Figure S12a, Supporting Information). The 1:4 sample shows larger short‐circuit current (*J*_SC_) than 1:1 for the broaden absorption region. The comparison between the 1:4 syn‐CsPbI_3_ PSCs with and without the DMAI surface passivation through tuning the annealing time is also conducted. Clearly, the sample with the DMAI surface passivation show larger open‐circuit (*V*_OC_) for the suppressing trap‐assisted nonradiative recombination at the interface, and hence greatly decreased its energy loss (Figure S12b, Supporting Information).

**Figure 6:**
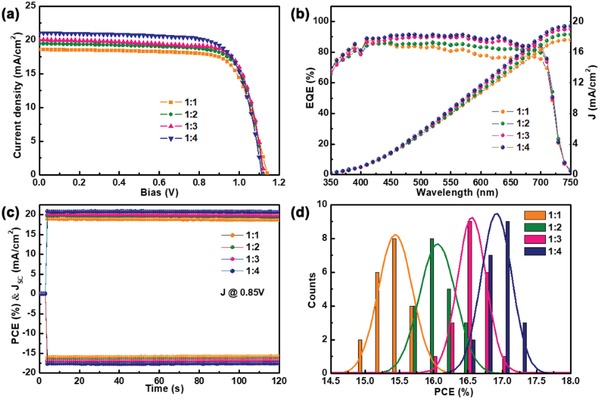
Photovoltaic performance of Syn‐CsPbI_3_ PSCs prepared using different HI ratios. a) *J*–*V* curves, b) EQE curves and the integrated products in an AM1.5G photon flux, c) stable photocurrent density and calculated efficiency established as a function of time for PSCs biased at 0.85 V, and d) device performance distributions for 20 cells. The curve represents the Gaussian fit of the histogram.

**Table 2:** Comparison of the device performance of different syn‐CsPbI_3_ films (extracted from Figure 6a)

Number	*J*_SC_ [mA cm^−2^]	*V*_OC_ [V]	FF [%]	PCE [%]	*J*_EQE_ [mA cm^−2^]
1:1	18.67 ± 0.18	1.13 ± 0.008	74.63 ± 0.66	15.6 ± 0.4	17.63
1:2	19.57 ± 0.26	1.12 ± 0.019	75.22 ± 0.69	16.3 ± 0.3	19.23
1:3	20.15 ± 0.17	1.10 ± 0.012	75.91 ± 1.01	16.7 ± 0.3	19.24
1:4	20.89 ± 0.26	1.08 ± 0.018	76.18 ± 0.78	17.0 ± 0.3	19.74

The corresponding external quantum efficiency (EQE) spectra of all devices exhibit a similar shape and negligible incompatibility between the integrated current and *J*_sc_ (Figure 6b). The stable photocurrent density of the PSCs is explored at the maximum power point (Figure 6c). Stable *J*_SC_ and PCEs are obtained for 120 s. Figure 6d reports reproducibility of the material and device fabrication. The PCE distribution of 20 individual devices has a fairly narrow distribution with good reproducibility.

The long‐term stability of the PSCs was examined under different exposure conditions, including nitrogen and air at relative humidity of 20–30%. As shown in Figure S13a in the Supporting Information, when the bare PSCs without any encapsulation was exposed to dry nitrogen at 80 °C, the PCE shows progressively degradation in 30 days. Noted that there is a certain increase in thermal stability as the HI content increasing. However, when the cell was exposed to high humidity after 7 days, PCE losses quickly as the absorber layer was transformed to its yellow phase. Similarly, the PCE exhibits gradually reduction as shown in Figure S13b in the Supporting Information, consistent with the N_2_ atmosphere. Clearly, excess HI addition to the precursor preparation can improve device stability, which might be due to the small amount of DMA^+^ doped into the CsPbI_3_ that adjusts its crystal structure.

In conclusion, we evaluated the impact of HI and DMF on the synthesis of stable CsPbI_3_. Characterization of XRD, TGA, and FTIR spectroscopy indicate that the previously reported “HI” additive and “HPbI_3_” consist of DMAI/DMAPbI*_x_*. Such intermediate compound improves the quality of the obtained perovskite film as well as the humidity stability and thermal stability. More importantly, the signal from the organic groups is very weak in the syn‐CsPbI_3_ film after annealing. Therefore, the main component of the syn‐CsPbI_3_ film prepared by this precursor is still inorganic as suggested by XPS and NMR with only ultrasmall amounts of doped DMA^+^ as Cs_1−_ *_x_*DMA*_x_*PbI_3_. Meanwhile, excessive DMA^+^ formed as Lewis acid–base adducts and interactions with Pb^2+^ on the CsPbI_3_ surface. As a result, the optimized CsPbI_3_ film‐based device shows a markedly enhanced stability in the ambient environment with a high PCE of 17.3%. The data show that this is a promising strategy to prepare high‐performance and air‐stable CsPbI_3_ PSCs.

## Conflict of Interest

The authors declare no conflict of interest.

## Supporting information

Supporting Information
