# Supplementary material for: Unveiling the Effects of Hydrolysis‐Derived DMAI/DMAPbIx Intermediate Compound on the Performance of CsPbI3 Solar Cells
Source: Adv Sci (Weinh). 2020 Mar 14;7(9):1902868. doi: 10.1002/advs.201902868 (PMC7201252; doi:10.1002/advs.201902868)
Supplement: Supplementary file 1 — Supporting Information [file ADVS-7-1902868-s001.pdf]

Copyright WILEY-VCH Verlag GmbH & Co. KGaA, 69469 Weinheim, Germany, 2019.

ADVANCED SCIENCE
Open Access

Supporting Information

for *Adv. Sci.,* DOI: 10.1002/advs.201902868

Unveiling the Effects of Hydrolysis-Derived DMAI/DMAPbI$_x$ Intermediate Compound on the Performance of CsPbI$_3$ Solar Cells

Hui Bian, Haoran Wang, Zhizai Li, Faguang Zhou, Youkui Xu, Hong Zhang, Qian Wang, Liming Ding, Shengzhong (Frank) Liu,* and Zhiwen Jin**

Supporting Information

Unveiling the Effects of Hydrolysis-Derived DMAI/DMAPbI$_x$ Intermediate Compound on Performance of $CsPbI_3$ Solar Cells

Hui Bian[1], Haoran Wang[1], Zhizai Li[2], Faguang Zhou[2], Youkui Xu[2], Hong Zhang[2,4], Qian Wang[2], Liming Ding[3,*], Shengzhong (Frank) Liu[1,*] and Zhiwen Jin[2,*]

Dr. H. Bian, Dr. H. Wang, Dr. Z. Li, Dr. F. Zhou, Dr. Y. Xu, Dr. H. Zhang, Prof. Q. Wang, Prof. L. Ding, Prof. S. Liu, Prof. Z. Jin
[1]Key Laboratory of Applied Surface and Colloid Chemistry, Ministry of Education; Shaanxi Key Laboratory for Advanced Energy Devices; Shaanxi Engineering Lab for Advanced Energy Technology; School of Materials Science & Engineering, Shaanxi Normal University, Xi'an 710119, PR China

[2]School of Physical Science and Technology & Key Laboratory for Magnetism and Magnetic Materials of MoE & Key Laboratory of Special Function Materials and Structure Design, MoE & National & Local Joint Engineering Laboratory for Optical Conversion Materials and Technology, Lanzhou University, Lanzhou 730000, China

[3]Center for Excellence in Nanoscience (CAS), Key Laboratory of Nanosystem and Hierarchical Fabrication (CAS), National Center for Nanoscience and Technology, Beijing 100190, China

[4]Electron Microscopy Centre, School of Physical Science and Technology, Lanzhou University, Lanzhou 730000, China.

E-mail: jinzw@lzu.edu.cn, ding@nanoctr.cn, szliu@dicp.ac.cn

Keywords: DMAPbI$_x$, DMAI, $CsPbI_3$, intermediate compound, solar cells

Experimental

Solution Preparation Section

Materials preparation: Dimethyl formamide (DMF), Dimethyl sulfoxide (DMSO), 4-tert-butylpyridine (TBP), bis (trifluoromethylsulfonyl) imidelithium salt (Li-TFSI), and chlorobenzene were purchased from Sigma-Aldrich. PbI_2, CsI, and $PbBr_2$ were purchased from Xi'an Polymer Light Technology Corp. All materials were used as received without further purification.

Precursor solution preparation: The syn-PbI_2 was prepared by using PbI_2 (1.2 g) dissolved in 2.5 mL DMF at 80 °C under active stirring for 30 min in air atmosphere. Immediately after, different volumes of HI (2.5 mL, 5 mL, 7.5 mL, 10 mL, 12.5 mL) were added to the solution for 2 h and dry overnight using a vacuum oven. Finally, 0.7022 g syn-PbI_2, 0.1468 g $PbBr_2$ and 0.3638 g CsI were added to the solution of 2 mL DMF and DMSO (9:1) under active stirring for 1 h. The $CsPbI_3$ precursor solutions were prepared. For comparison, we prepared the same ratio of solutions using PbI_2 purchased directly.

HTL solution preparation: A solution was prepared by dissolving PTAA (36 mg), a sulfonyl imide (Li-TFSI, 22 μL, 520 mg Li-TFSI in 1 mL acetonitrile), and tert-butylpyridine (TBP, 36 μL) in 1 mL of CB solution.

Device Fabrication Section

Preparation of TiO_2-blocking layer: FTO-coated glass was washed and O_2-plasma-treated for 15 min before the deposition of TiO_2. The clean substrates were immersed in a 40 mM $TiCl_4$ aqueous solution for 1h at 70 °C and washed with distilled water and ethanol, followed by annealing at 200 °C for 30 min in air to form a compact TiO_2 layer.

Growth of the $CsPbI_3$ film: The $CsPbI_3$ layer was fabricated via one-step spin-coating. The mixed solution was spin-coated on top of O_2-plasma-treated TiO_2 substrate at 1000 rpm for 10 s. The speed was then increased to 3500 rpm and maintained 40 s. Finally, the films were annealed at 180 °C for 15mim to form the films.

Assembly of the solar cells: An HTL film was prepared by spin-coating the HTL solution onto the $CsPbI_3$ film at 5000 rpm for 30 s. A 70-nm-thick gold electrode was then thermally evaporated onto the HTL-coated film.

Characterization Section

SEM and AFM: The film surface morphology, elemental distribution, and cross-sectional images were characterized by field emission scanning electron microscope (FESEM, Jeol SU-8020). Atomic force microscope (AFM, Bruker Dimension 5000 Scanning Probe Microscope) was used to measure surface roughness of the perovskite film in "tapping" mode.

Absorbance and PL: Absorbance spectra were collected using a Shimadzu UV-3600 double beam spectrometer using the slowest scanning rate with one-second integration and a 2 nm slit width. The PL spectra were measured using a PicoQuant FluoTime 300.

The source light was a xenon short arc lamp. PL (excitation at 532 nm) spectra were measured using a FLS980 spectrometer (Edinburgh Instruments Ltd)

XRD, FTIR, NMR and XPS: XRD patterns of the samples were obtained using a Bruker D8 GADDS Diffractometer with the Cu Kα line. FTIR spectra were measured with a Bruker Vertex 70. NMR was performed by using JNM-ECZ400S/L1 with a frequency of 400 MHz and deuterated DMSO was used as solvent. The XPS measurements were performed in a VG ESCALAB MK2 system with monochromatized Al Kα radiation at a pressure of 5.0×10^{-7} Pa. XPS was carried out by using a photoelectron spectrometer (ESCALAB250Xi, Thermo Fisher Scientifc).

J-V and EQE: The active area of solar cell was restrained by a 9 mm^2 metal mask. The J–V characteristics of the PSCs were collected by using a digital source meter (Keithley Model 2400) under an illumination of an AM 1.5 solar simulator (100 mW cm^{-2}, SS-F5-3A, Enlitech), as calibrated by a NREL-traceable KG5-fltered silicon reference cell. This used reverse scan mode (from V_{OC} to I_{SC}) and forward scan mode (from I_{SC} to V_{OC}) with a scan rate of 30 mV/s. The external quantum efficiency (EQE) data were obtained by solar-cell spectral-response measurement system (QE-R3011, Enlitech). The monochromatic light intensity for EQE was calibrated using a reference silicon detector.

Long-term stability analysis: The steady photocurrent and PCE were measured at the maximum power point. The long-term stability was measured after storage in air (relative humidity of 30%-40% at 25 °C) over 7 days without any encapsulation. To test the device heat stability, we put the unencapsulated device in a nitrogen glovebox with 80 °C for 30 days.

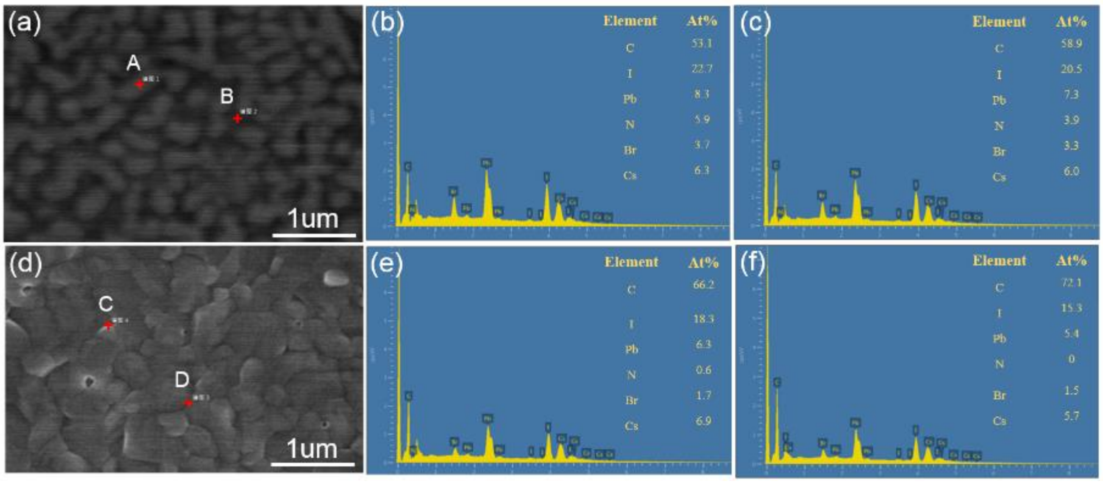

Figure S1. Characterization of the Syn-$CsPbI_3$ Film (a) The SEM image of unannealed syn-$CsPbI_3$ film. (b) and (c) are the EDX spectra of points A and B in **Figure S1a**, respectively. (d) The SEM image of annealed syn-$CsPbI_3$ film. (e) and (f) are the EDX spectra of points C and D in **Figure S1d**, respectively.

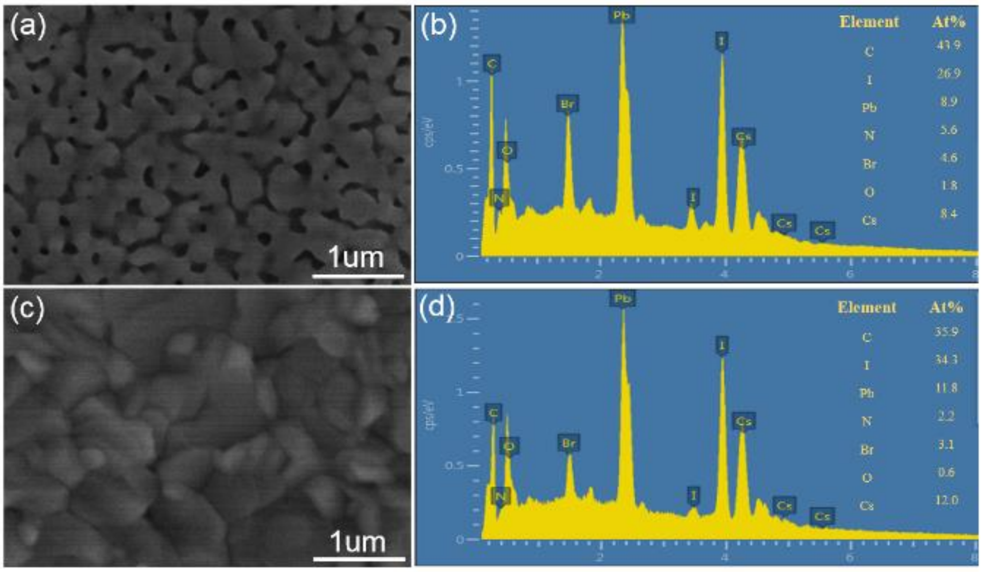

Figure S2. Characterization of the Syn-$CsPbI_3$ Film. The SEM image and EDX spectra of (a)-(b) unannealed syn-$CsPbI_3$ film. (c)-(d) annealed syn-$CsPbI_3$ film.

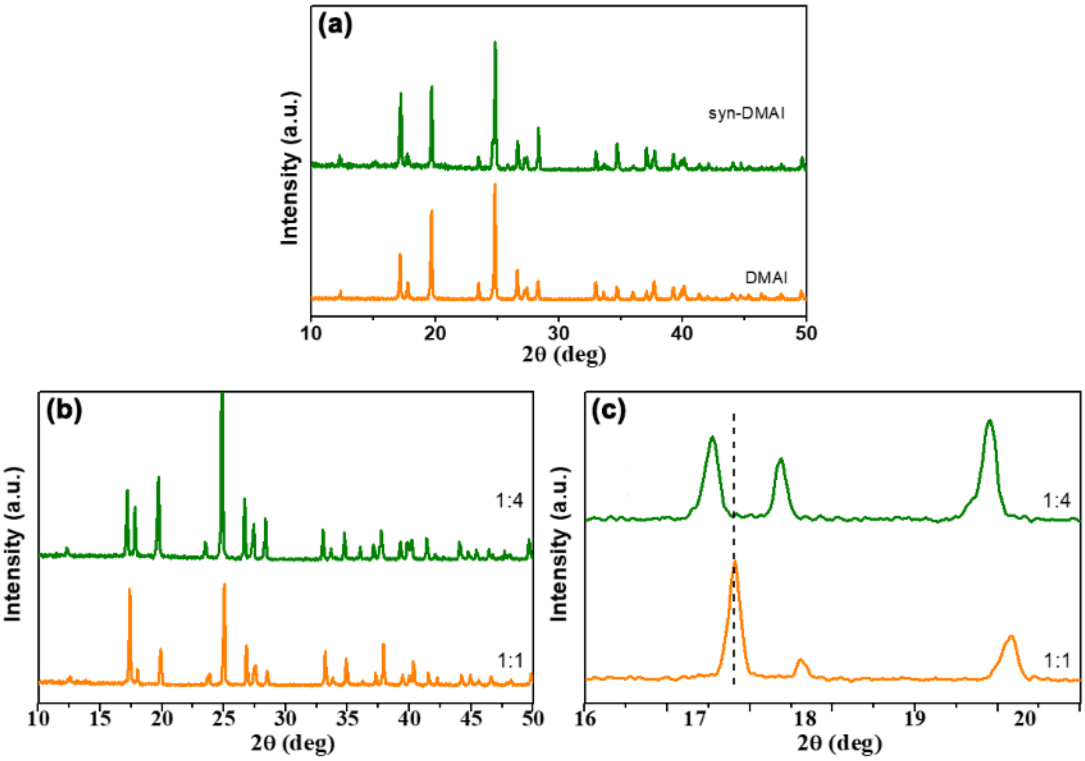

Figure S3. Characterization of Syn-DMAI Powder. (a) XRD patterns of purchased DMAI and syn-DMAI; (b) XRD patterns of syn-DMAI using different HI ratios; (c) corresponding magnification of a portion of the XRD patterns.

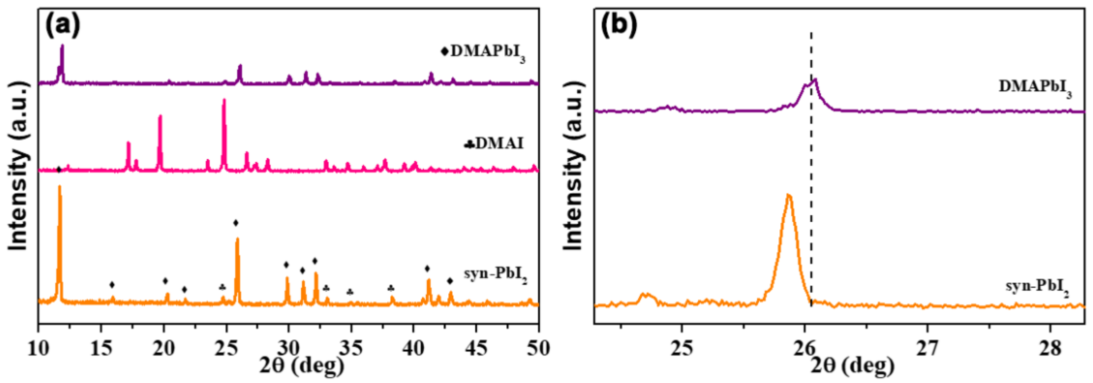

Figure S4. Films and Powders Component Studies. XRD patterns of (a) the syn-PbI_2 powder, DMAI and $DMAPbI_3$; (b) the corresponding magnification of a portion for syn-PbI_2 powder and $DMAPbI_3$.

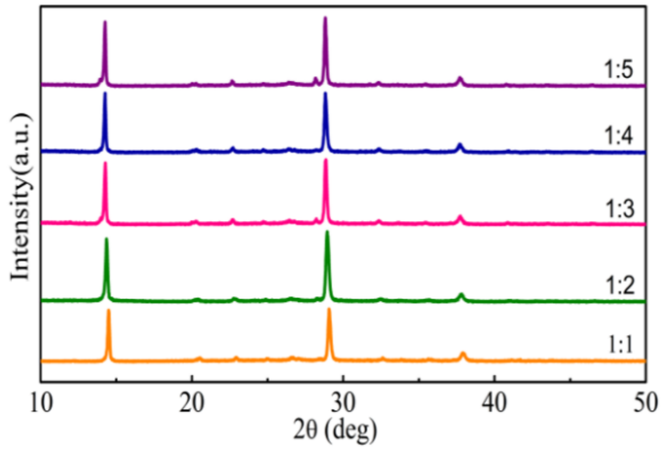

Sample	FWHM
1:1	0.21
1:2	0.22
1:3	0.17
1:4	0.17
1:5	0.16

Figure S5. XRD patterns of syn-$CsPbI_3$ films and the corresponding FWHM of the peaks (110).

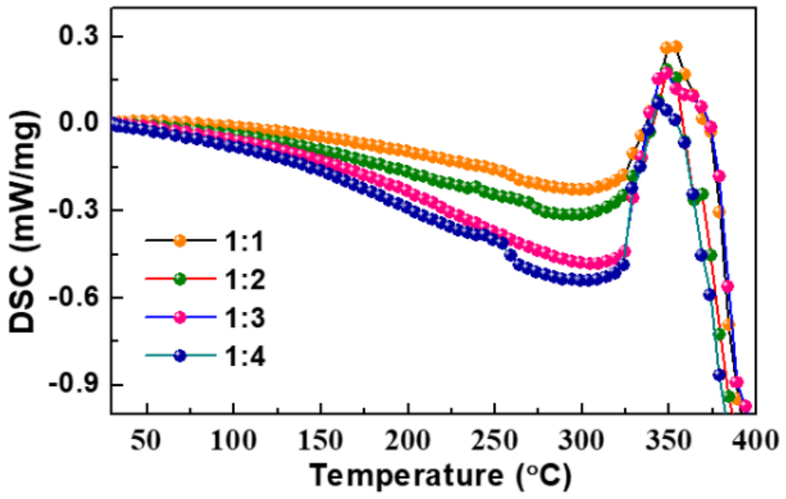

Figure S6. DSC curves of Syn-PbI_2 Powder.

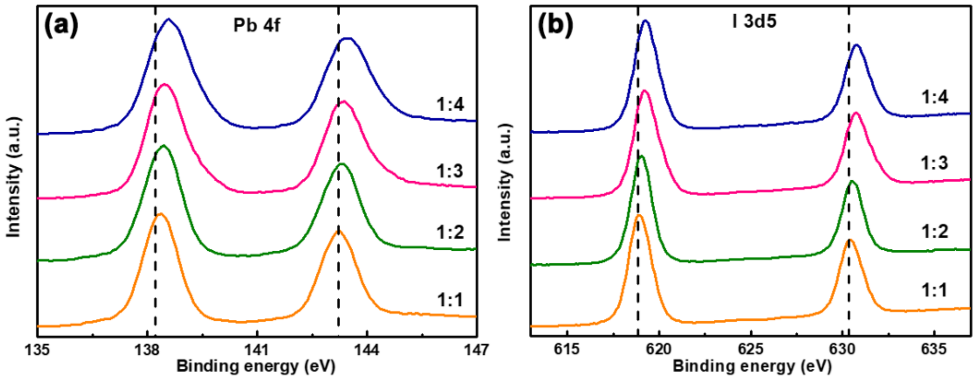

Figure S7. XPS spectra of (a) Pb 4f; (b) I 3d5 in syn-PbI_2 powder.

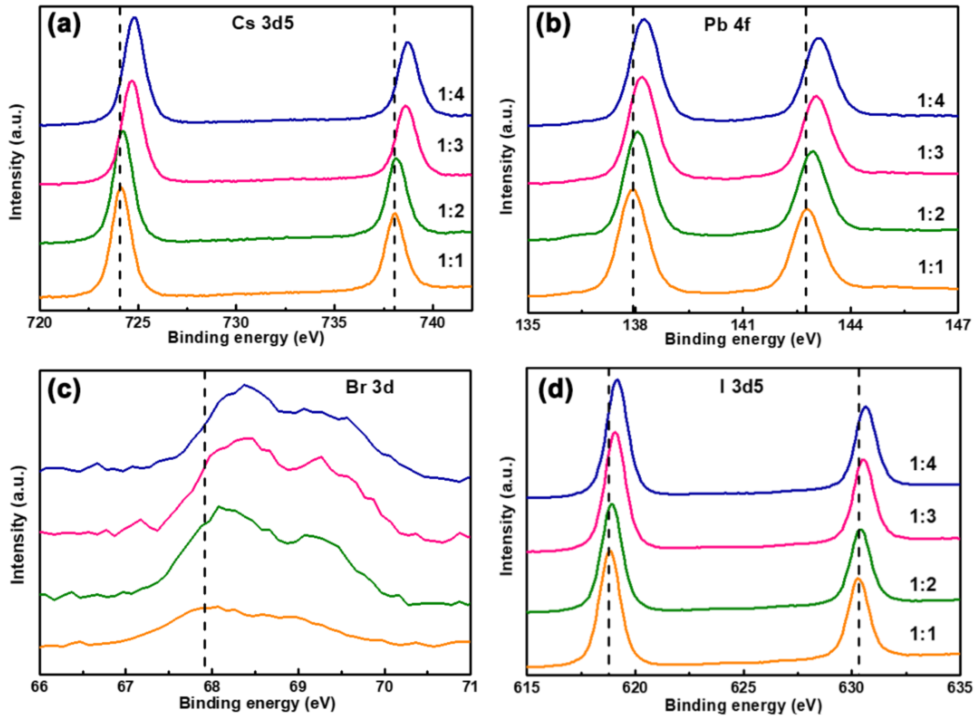

Figure S8. XPS spectra of (a) Cs 3d5; (b) Pb 4f; (c) Br 3d; and (d) I 3d5 in syn-$CsPbI_3$ film.

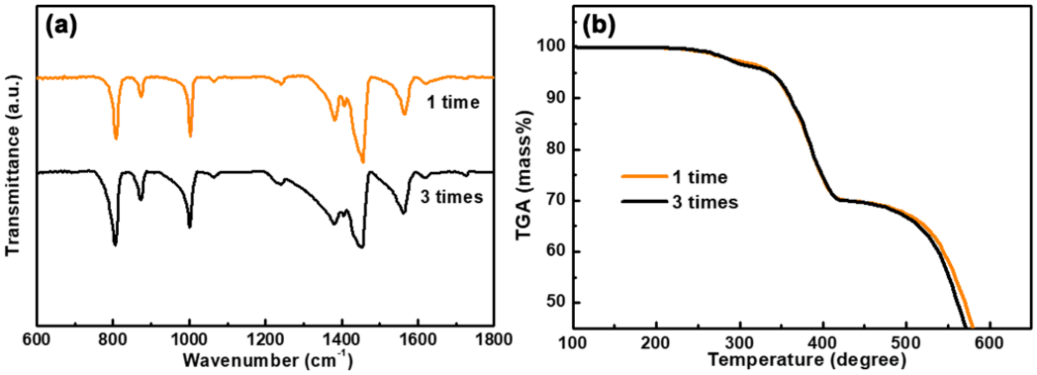

Figure S9. Different purification times for syn-PbI_2: (a) FTIR spectra; (b) TGA curves.

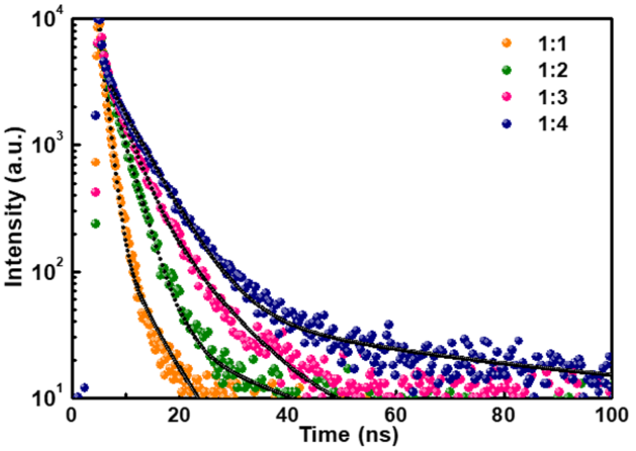

Figure S10. TRPL decay curves for syn-$CsPbI_3$ films.

Table S1. TRPL spectroscopy fitting parameters of the syn-$CsPbI_3$ films (extracted from **Figure S10**).

Sample	τ_{ave} (ns)	τ_1 (ns)	amplitude τ_1 (%)	τ_2 (ns)	amplitude τ_2 (%)
**1:1**	1.56	5.63	11.15	1.05	88.85
**1:2**	4.18	18.76	8.85	2.76	91.15
**1:3**	4.76	8.9	29.25	3.04	70.75
**1:4**	10.87	48.01	12.74	5.45	87.26

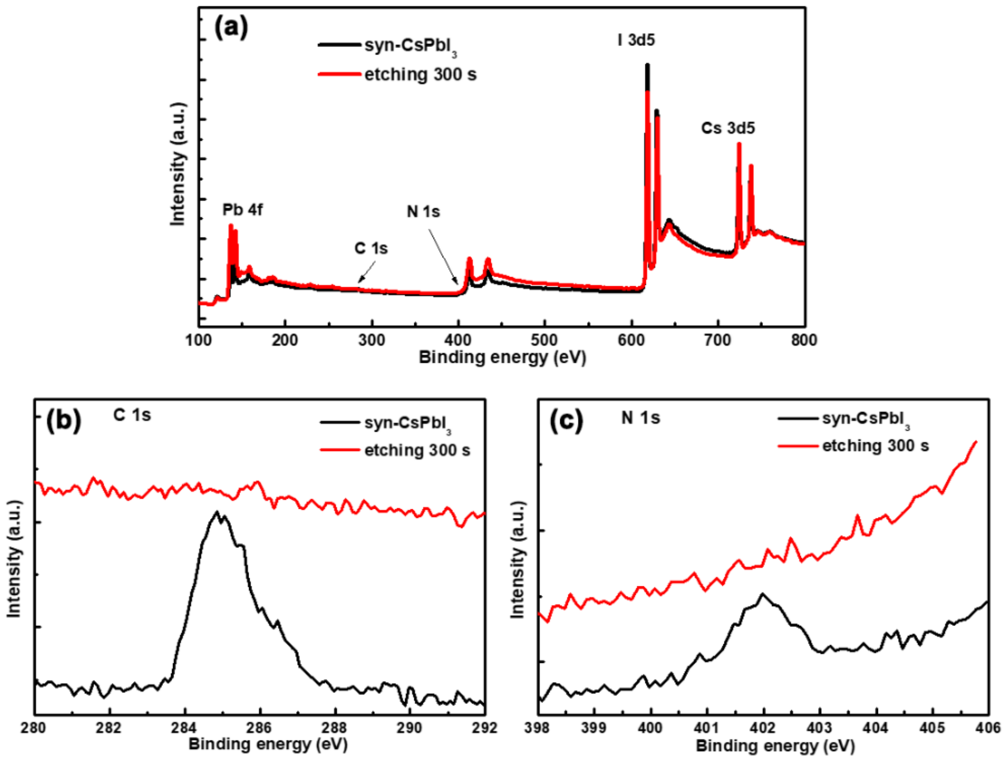

Figure S11. (a) XPS spectra of syn-$CsPbI_3$ film before and after etching 300 s; (b) C 1s; (c) N 1s.

Table S2. The measured elements value of the XPS spectra of syn-$CsPbI_3$ film before and after etching 300 s (extracted from **Figure S11a**).

Element	C	I	Pb	N	Br	O	Cs
At% (0nm)	18.73	43.6	12.94	0.07	3.88	2.19	18.59
At% (0nm)	0	46.85	20.48	0	8.1	0	24.57

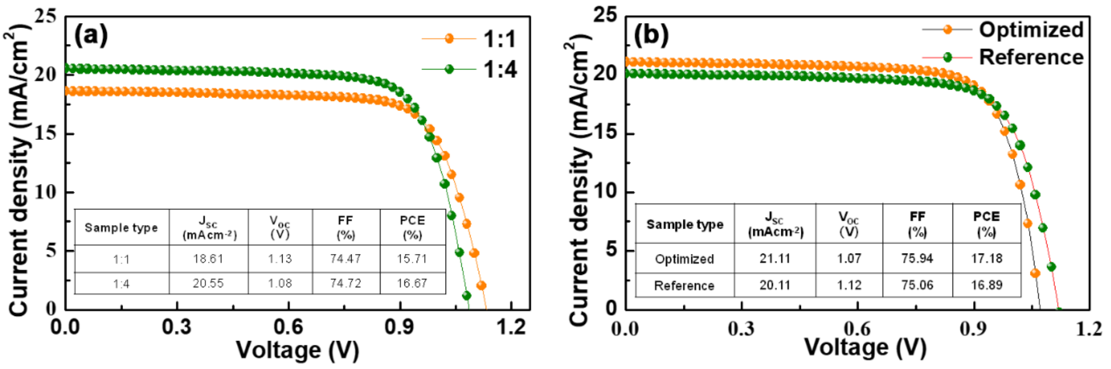

Figure S12. Photovoltaic Performance of Syn-$CsPbI_3$ PSCs. (a) J-V curves of 1:1 and 1:4 samples after longer annealing process; (b) J-V curves for 1:4 sample of optimized device and reference device with longer annealing process.

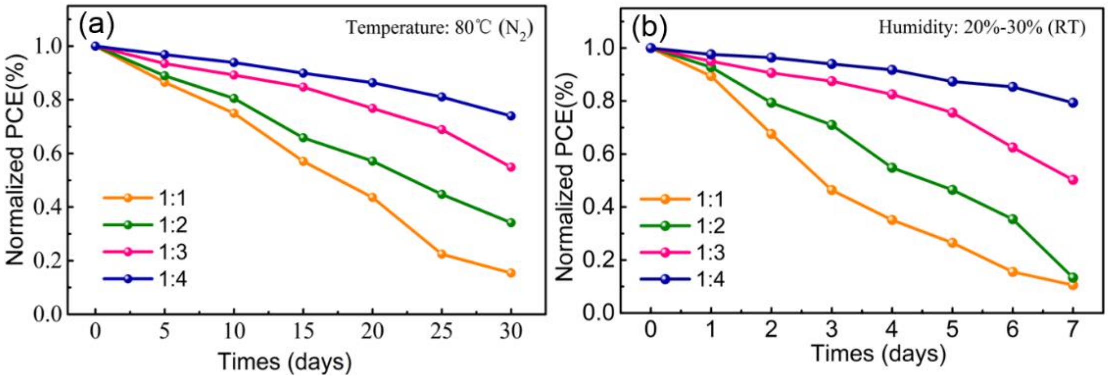

Figure S13. Long-term stability of the best-performing device stored: (a) in N_2 atmosphere at 80 °C; (b) in air at relative humidity of 20%-30%.
